# Pharmacological Cyclophilin Inhibitors Prevent Intoxication of Mammalian Cells with *Bordetella pertussis* Toxin

**DOI:** 10.3390/toxins10050181

**Published:** 2018-05-01

**Authors:** Katharina Ernst, Nina Eberhardt, Ann-Katrin Mittler, Michael Sonnabend, Anna Anastasia, Simon Freisinger, Cordelia Schiene-Fischer, Miroslav Malešević, Holger Barth

**Affiliations:** 1 Institute of Pharmacology and Toxicology, University of Ulm Medical Center, 89081 Ulm, Germany; nina.eberhardt@uni-ulm.de (N.E.); ann-katrin.mittler@uni-ulm.de (A.-K.M.); michael.sonnabend@web.de (M.S.); anna.anastasia@uni-ulm.de (A.A.); simon.freisinger@uni-ulm.de (S.F.); 2 Institute for Biochemistry and Biotechnology, Martin Luther University Halle-Wittenberg, 06120 Halle (Saale), Germany; cordelia.schiene-fischer@biochemtech.uni-halle.de (C.S.F.); miroslavmalesevic1@gmail.com (M.M.)

**Keywords:** pertussis toxin, whooping cough, cellular uptake, membrane transport, cyclosporine A, cyclophilins, chaperones, novel drug targets

## Abstract

The *Bordetella pertussis* toxin (PT) is one important virulence factor causing the severe childhood disease whooping cough which still accounted for approximately 63,000 deaths worldwide in children in 2013. PT consists of PTS1, the enzymatically active (A) subunit and a non-covalently linked pentameric binding/transport (B) subunit. After endocytosis, PT takes a retrograde route to the endoplasmic reticulum (ER), where PTS1 is released into the cytosol. In the cytosol, PTS1 ADP-ribosylates inhibitory alpha subunits of trimeric GTP-binding proteins (Giα) leading to increased cAMP levels and disturbed signalling. Here, we show that the cyclophilin (Cyp) isoforms CypA and Cyp40 directly interact with PTS1 in vitro and that Cyp inhibitors cyclosporine A (CsA) and its tailored non-immunosuppressive derivative VK112 both inhibit intoxication of CHO-K1 cells with PT, as analysed in a morphology-based assay. Moreover, in cells treated with PT in the presence of CsA, the amount of ADP-ribosylated Giα was significantly reduced and less PTS1 was detected in the cytosol compared to cells treated with PT only. The results suggest that the uptake of PTS1 into the cytosol requires Cyps. Therefore, CsA/VK112 represent promising candidates for novel therapeutic strategies acting on the toxin level to prevent the severe, life-threatening symptoms caused by PT.

## 1. Introduction

The *Bordetella (B.) pertussis* toxin (PT) is a multi-subunit protein toxin consisting of an enzymatically active (A) subunit, namely PTS1, which is non-covalently associated with a pentameric binding/transport (B) subunit [1,2]. Therefore, PT is classified as an AB_5_ toxin. The B subunit is formed by the S2, S3, two S4 and the S5 proteins. The holotoxin is assembled in the periplasm of *B. pertussis* and then secreted by a type IV secretion system [3,4]. PT binds to glycoconjugate molecules on its target cells. A specific receptor, however, is not known, instead the binding of PT is characterized as non-saturable and non-specific [5,6,7]. PT is internalized by endocytosis and then follows a retrograde transport passing the Golgi apparatus towards the endoplasmic reticulum (ER). The lactone antibiotic brefeldin A (BFA) inhibits vesicle formation as well as transport between ER and Golgi apparatus in cells and therefore protects cells from intoxication with PT [8,9,10,11]. In the ER, PTS1 is detached from the B pen tamer after the binding of ATP to the central ‘pore’ of the B oligomer [12,13,14]. Due to its thermal instability, the detached PTS1 is in an unfolded conformation, which makes it a substrate for the ER-associated degradation (ERAD) pathway, which transports PTS1 from the ER into the cytosol [15,16,17]. The subsequent ubiquitin-dependent degradation by the proteasome is circumvented because PTS1 does not contain lysine residues, which are required for ubiquitination of proteins [18]. In the cytosol, PTS1 mediates the covalent transfer of an ADP-ribose moiety from the co-substrate NAD^+^ onto its specific substrate, the α-subunit of trimeric inhibitory GTP-binding proteins (Giα), which results in inactivation of Giα [19,20]. Because Giα normally serves as a negative regulator of a membrane-bound adenylate cyclase, the PTS1-catalyzed modification in return results in increased intracellular cAMP levels and disturbed signal-transduction in PT-intoxicated cells.

PT plays an etiological role in causing whooping cough and promotes a more severe course of disease [21,22]. Whooping cough is characterized by severe paroxysmal coughing typically lasting for several weeks. Secondary complications include vomiting, rib fractures and pneumothorax and in severe cases whooping cough can be life-threatening especially for newborns and infants due to pneumonia, encephalopathy, seizures and apnoea [23,24]. The world health organization (WHO) reported estimates of 63,000 deaths in children aged < 5 years in 2013 worldwide caused by whooping cough with numbers increasing despite available vaccination [25,26].

Up to now, there is no causative treatment of whooping cough that targets the disease on the toxin level. Antibiotics are applied to prevent spreading of the disease but is only effective if administered in the early stage of infection and has no curative effect on the severe symptoms [23,24]. Therefore and because severe life-threatening courses of whooping cough are associated with high levels of PT, novel therapeutic approaches are demanded that act specifically on the toxin level.

Here, we investigate the effect of cyclosporine A (CsA), an approved immunosuppressive drug mostly applied after organ transplantation, on intoxication of Chinese hamster ovary (CHO)-K1 cells with PT. CsA is a specific pharmacological inhibitor of cyclophilin (Cyps) activity in cells. Cyps are important protein folding helper enzymes that catalyse the peptidyl-prolyl *cis/trans* isomerization representing a rate-limiting step in protein folding. Earlier we reported that Cyps are required to facilitate the membrane translocation from early endosomes into the cytosol of clostridial binary toxins, diphtheria toxin and *Photorhabdus luminescens* PTC3 toxin, which also display ADP-ribosyltransferase activity [27,28,29,30,31]. Inhibition of Cyps by CsA inhibited the membrane translocation of the A subunits of such toxins and thereby protected cells from intoxication with these ADP-ribosylating toxins (ADP-RTs), prompting the question of whether CsA might also protect cells from intoxication with PT. In conclusion, the results show that PTS1 directly binds to purified Cyp proteins in vitro and that the pharmacological inhibition of Cyp activity protects CHO-K1 cells from intoxication with PT.

## 2. Results

Specific pharmacological inhibition of Cyp activity protects cells from intoxication with PT. To investigate an inhibitory effect of CsA on PT intoxication of cells, an intoxication assay was performed, which employs a morphology-based read out of CHO-K1 cells. CHO-K1 cells respond to treatment with PT by a characteristic clustering and a reduced cell number compared to untreated cells. Although the molecular mechanisms underlying this clustering are not understood, the clustering effect represents a specific and sensitive endpoint of PT intoxication since it can be prevented by anti-PT antibodies or heat inactivation of PT [32].

**Figure 1:**
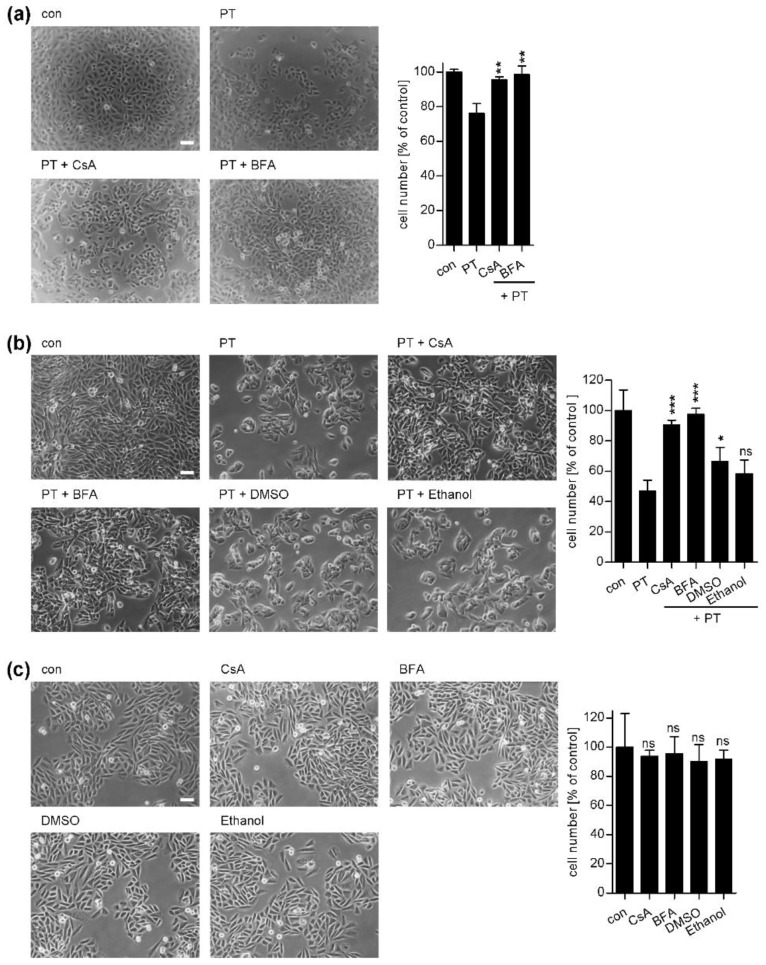
(**a**) Effect of cyclosporine A (CsA) on intoxication of non-adherent CHO-K1 cells with *Bordetella pertussis* toxin (PT). CHO-K1 cells were pre-incubated with 10 µM CsA or brefeldin A (BFA) or left untreated for control. After 30 min, the cells were trypsinized and seeded in 96-well plates with culture medium containing 10 ng/mL PT or no toxin for control and incubated at 37 °C and 5% CO_2_ without inhibitors. After 18 h of incubation, the cells were fixed with PFA and pictures were taken (left panel, white scale bar = 50 µm). Right panel: A quantitative analysis of the total cell number of CHO-K1 cells from an equivalent experiment without PFA treatment is shown. Values are normalized on control cells (*n* = 3 (3 independent wells per treatment, one picture per well), mean ± SD). (**b**) Effect of CsA on the intoxication of adherent CHO-K1 cells with PT. CHO-K1 cells were pre-incubated with 10 µM CsA or BFA or left untreated for control. For further control, cells were incubated with DMSO or ethanol, the solvent of BFA and CsA, respectively. The final concentration of DMSO in the medium was 2-fold higher compared to the DMSO concentration used in all inhibitor experiments. After 30 min 10 ng/mL PT were added. 1 h later the culture medium was removed and the cells were further incubated at 37 °C and 5% CO_2_ in fresh medium without PT and inhibitors. Pictures were taken after 18 h (left panel, white scale bar = 50 µm). Right panel: A quantitative analysis of the total cell number of CHO-K1 cells is shown, values are normalized on control cells (*n* = 3 (3 different pictures from each sample were quantified), mean ± SD). (**c**) Effect of CsA, BFA DMSO or ethanol on CHO-K1 cell numbers. Cells were incubated with 10 µM CsA or BFA, DMSO (amount corresponding to the 2-fold maximum amount used for inhibitor incubation) or ethanol for 1 h 45 min. Medium was removed and fresh medium without inhibitors was added for 18 h. Pictures were taken (left panel, white scale bar = 50 µm) and cell numbers determined as described above (right panel). Significance was tested by using the Student’s t test and values refer to samples treated with toxin only (**a**,**b**) or to control samples (**c**) (ns = not significant, * *p* < 0.05, ** *p* < 0.01, *** *p* < 0.001).

This well-established assay was performed earlier by several groups and different variants of this assay were used to monitor intoxication of these cells with PT [32,33]. Here, we tested two variants of this assay. For the first variant, CHO-K1 cells were pre-incubated with the respective inhibitors, then cells were detached using trypsin and reseeded into a 96-well plate containing medium with PT for 18 h [33]. Pictures in Figure 1a show the typical clustering morphology in PT-treated samples and the quantification of cells from these pictures clearly demonstrates the reduced cell number in samples treated with PT. In this assay variant, the PT-induced effect on cell morphology was obvious, however the magnitude was considered to be improvable. Therefore, we employed a different assay variant using adherent CHO-K1 cells which were pre-incubated with inhibitors and subsequently treated with PT for 1 h. Then, the cells were further incubated for 18 h with medium containing no toxin or inhibitors because obvious adverse effects on the cells such as shrinking and rounding were observed if inhibitors were present for the extended incubation period of 18 h. Under these conditions, the clustering effect was much more pronounced and also the effect on cell numbers was more obvious (Figure 1b). Therefore, this assay variant was used for further experiments. However, an inhibitory effect of CsA on intoxication of CHO-K1 cells with PT was observed in both assay variants (Figure 1a,b) demonstrated by reduced clustering and increased cell numbers compared to samples treated with PT only. Noteworthy, the presence of CsA in the medium during the pre-incubation and subsequent incubation with PT for 1 h was sufficient to protect cells from intoxication. Due to its specific properties with respect to binding to its intracellular partner proteins, the Cyps but also due to the lipophilicity of the CsA molecule, there is only a slow loss of intracellular CsA over time in the absence of extracellular CsA. This is concomitant with the long retention of CsA in different tissues and the long plasma terminal half-life values (20–60 h) observed after CsA administration in human patients [34,35]. Moreover, in the case of HEK293T cells Shitara et al. showed that after the removal of CsA from the incubation buffer and further incubation in CsA-free medium, the amount of CsA in the cells was decreased but more than 50% remained in the cells for 18 h [36]. Thus, presumably intracellular Cyps stay inhibited over a longer period of time explaining the toxin-resistant phenotype. In both assay variants, the established inhibitor BFA, which leads to disassembly of the Golgi apparatus, was used as a positive control and protected CHO-K1 cells from intoxication with PT [8,11]. Although reversibility of the BFA-effect is known if this compound is removed from the culture medium, here too, pre-incubation and presence of BFA during toxin incubation was sufficient to achieve a protective effect towards PT. As a further control, DMSO and ethanol, the solvent of BFA and CsA, respectively, had no inhibitory effect on the intoxication process (Figure 1b). CsA, BFA, DMSO and ethanol did not affect the cell numbers under assay conditions (Figure 1c).

**Figure 2:**
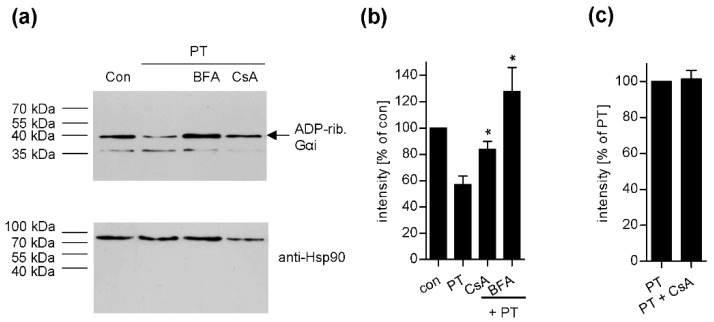
Effect of CsA on the ADP-ribosylation status of Giα in PT-treated CHO-K1 cells. (**a**) Cells were pre-incubated with 20 µM of CsA or 20 µM BFA for control for 30 min. For further control, cells were left untreated. Then, cells were challenged with 20 ng/mL PT for 3 h in the presence or absence of the respective inhibitors. Cells were lysed and the ADP-ribosylation status of Giα from these cells was analysed by incubation with 170 ng PTS1 in the presence of 10 µM biotin-labelled NAD^+^. Biotin-labelled (i.e., ADP-ribosylated) Giα was detected with streptavidin-peroxidase. Comparable protein loading was confirmed by Ponceau-S-staining (not shown) and Hsp90 detection by Western blotting. (**b**) Intensity of signals for biotinylated Giα detected by Western blotting were quantified by densitometry. Values show the percentage of signal intensity referring to untreated cells (Con) and are normalized on the respective loading control obtained by Ponceau S staining. Values are given as mean ± SEM (n = 4 independent experiments). Significance was tested by using the Student’s t test and values refer to samples treated with toxin only (* *p* < 0.05). (**c**) CsA does not affect binding of PT to cells. CHO-K1 cells were pre-incubated with 20 µM CsA for 30 min or left untreated for control. Subsequently, cells were incubated on ice with 500 ng/mL PT for 40 min and then washed twice with PBS. Samples were subjected to SDS-PAGE followed by Western Blotting. PTS1 was detected by a specific monoclonal antibody using the ECL system. Signals were quantified by densitometry and are given as percent of the sample treated with PT only. Values are given as mean ± SD (*n* = 3 values from 2 independent experiments).

Inhibition of Cyp activity in cells affects the ADP-ribosylation status of Giα in the cytosol. The analysis of the ADP-ribosylation status of specific substrates in target cells is a further endpoint of intoxication established for many ADP-RTs like diphtheria toxin or clostridial binary toxins [27,31]. Here, CHO-K1 cells were intoxicated with PT in the presence or absence of CsA or BFA and subsequently lysed. Cell lysates were incubated with fresh PTS1 in the presence of biotin-labelled NAD^+^ allowing the in vitro ADP-ribosylation of Giα that was not already modified during the incubation of the living cells, thereby transferring the biotin-labelled ADP-ribose moiety covalently onto Giα. Therefore, the amount of biotin-labelled Giα detected by Western blotting indicates the degree of intoxication of cells with PT. Figure 2a,b reveals that samples treated with PT result in a weak signal for biotin-labelled Giα compared to untreated controls. This indicates that only a small amount of Giα serves as substrate for in vitro ADP-ribosylation because the substantial amount of Giα was already ADP-ribosylated during incubation of the living cells with PT. If the cells were pre-incubated with CsA prior to PT application, a stronger signal for biotin-labelled Giα was detected. This result demonstrates that CsA protected the living cells from intoxication with PT. A similar result was observed when cells were treated with BFA prior to PT intoxication, confirming the specificity of this method. Moreover, we excluded that the reduced ADP-ribosylation of Giα by PTS1 in the presence of CsA results from impaired binding of PT to cells. Figure 2c demonstrates that a comparable amount of cell-associated PTS1 was detected by Western blotting if cells were treated with CsA compared to cells treated with PT alone.

**Figure 3:**
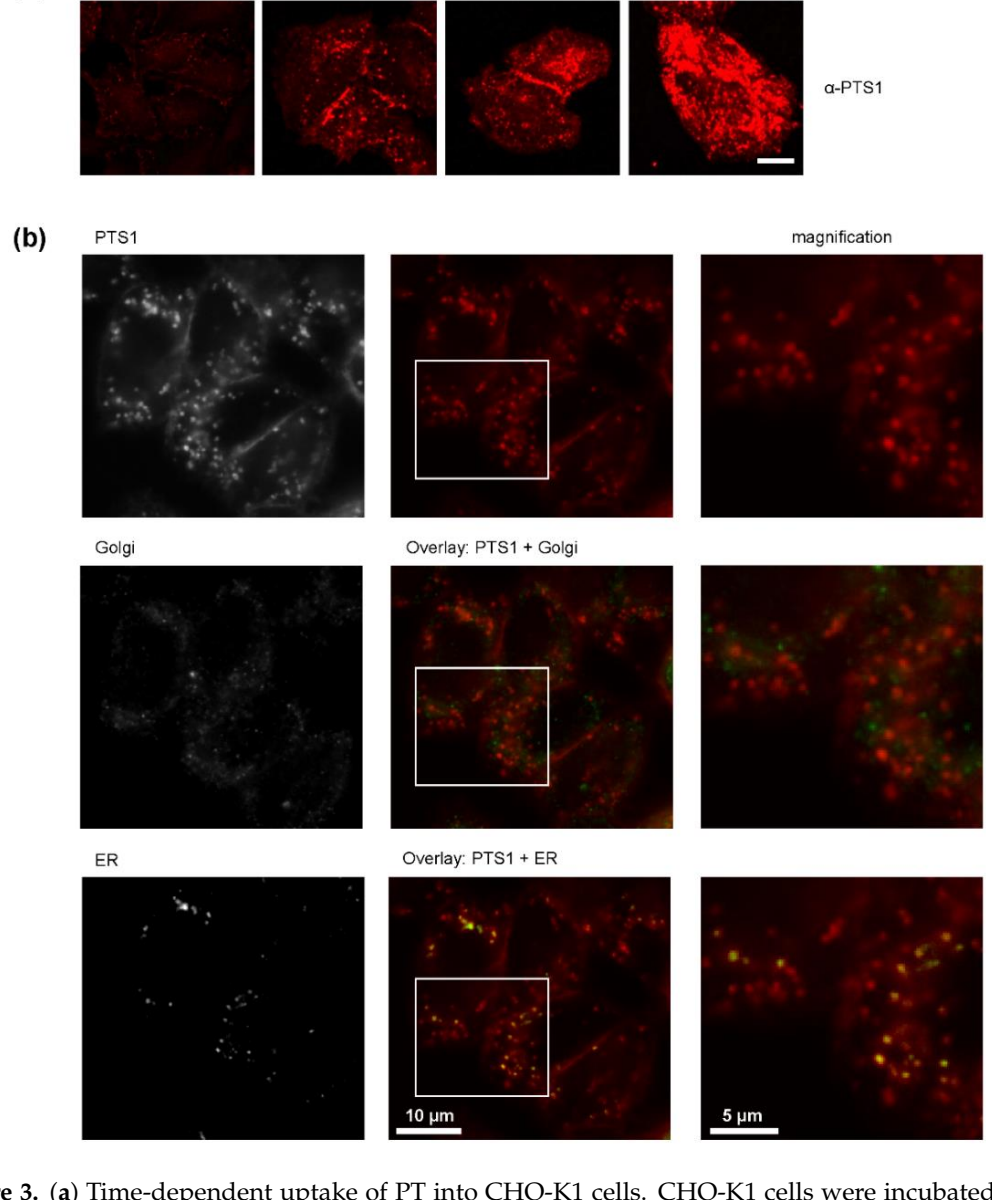
(**a**) Time-dependent uptake of PT into CHO-K1 cells. CHO-K1 cells were incubated with PT (1 µg/mL). After the indicated time points, cells were fixed, permeabilized and blocked. PTS1 was detected with a specific monoclonal antibody. Pictures were taken with a Zeiss LSM-710 confocal microscope. Scale bar = 10 µm. (**b**) Co-staining of PTS1 with markers for Golgi or ER. CHO-K1 cells were incubated with PT (1 µg/mL) in the presence of ER-Tracker^TM^ Blue White DPX (1 µM) for 3 h. Cells were fixed, permeabilized and blocked. PTS1 and GM-130 (Golgi marker) were detected by specific antibodies allowing the simultaneous detection of ER, Golgi and PTS1 in one sample. Left row of pictures shows the signal for the three single channels detected, middle row shows the PTS1 signal alone or in overlay with either Golgi or ER and the right row shows a magnification of the areas indicated by the white rectangle.

In the presence of CsA less PTS1 is detectable in cells. The obtained results suggested that the pharmacological inhibition of Cyp activity interferes with the uptake of PTS1 into the cytosol of target cells, which is a crucial point for intoxication since the toxin’s substrate Giα is localized in the cytosol. To analyse the amount of PTS1 that reaches the cytosol of target cells, we performed fluorescence microscopy visualizing PTS1. First, CHO-K1 cells were incubated with PT for different periods to establish after which incubation periods PTS1 can be detected in the cells by immunofluorescence microscopy. The results shown in Figure 3a revealed that PTS1 was already detected after 5 min and continuous detection was possible after 30 min, 1.5 h and 24 h of incubation of the cells with PT. Moreover, co-staining of PTS1 with markers for Golgi and ER was performed. Figure 3b demonstrates that PTS1 showed almost no co-localization with the Golgi marker but partial co-localization with the ER marker. However, a substantial amount of PTS1 did not show co-staining with the ER marker, suggesting that this portion is most likely cytosolic PTS1.

**Figure 4:**
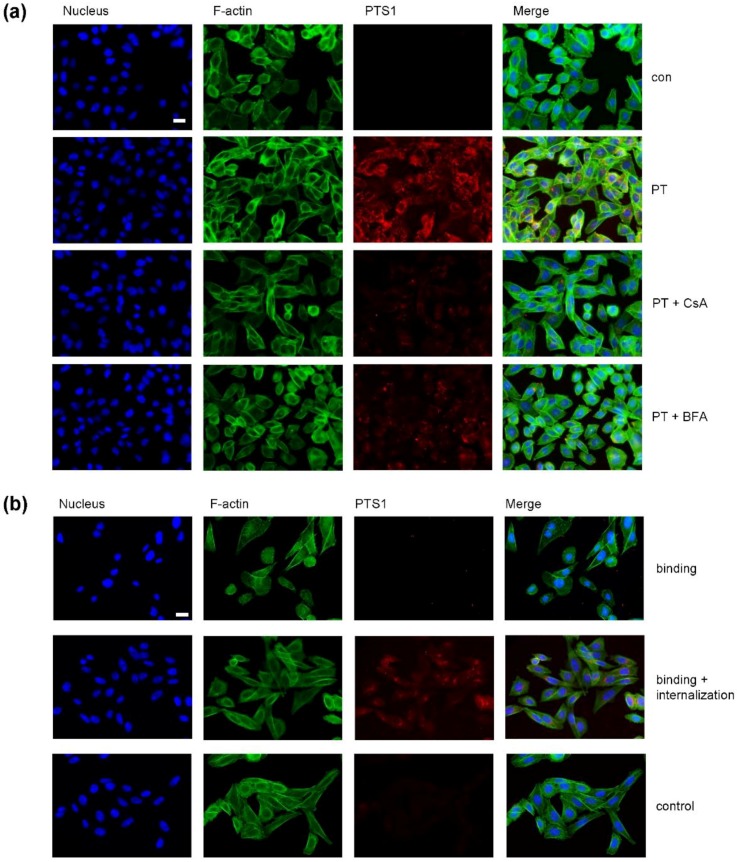
(**a**) In the presence of CsA less PTS1 is detected in CHO-K1 cells. CHO-K1 cells were pre-incubated with CsA (20 µM) for 30 min. 20 µM BFA were used as control. Then, cells were challenged with 1 µg/mL PT in the presence or absence of the respective inhibitors and 3 h later cells were fixed, permeabilized and blocked. Then, cells were probed with an anti-PTS1 antibody, Hoechst and phalloidin-FITC for F-actin staining. Scale bar = 20 µm. (**b**) Anti-PTS1 antibody does not detect cell-bound PTS1. CHO-K1 cells were incubated on ice for 10 min and then challenged with 1 µg/mL PT for 30 min to enable only the binding to the cells. For control, cells were left untreated. Subsequently, cells were washed three times with PBS to remove unbound toxin. Then one portion of PT-treated cells was fixed immediately with PFA and another portion was further incubated at 37 °C for 2 h with subsequent PFA fixation. All samples were permeabilized, blocked and probed with a specific PTS1-antibody and a fluorescence-labelled secondary antibody. Nuclei were stained with Hoechst and F-actin with phalloidin-FITC. Scale bar = 20 µm.

The PTS1 signal significantly decreased if cells were incubated with CsA (Figure 4a) compared to cells challenged with PT in the absence of CsA. This effect was also observed if the transport of PT from the Golgi apparatus to the ER was inhibited by BFA. Moreover, PTS1 could not be detected by the specific monoclonal PTS1 antibody on the cell surface if cells were incubated with PT on ice (Figure 4b, upper panel). To confirm that PT indeed binds to cells at 4 °C, another sample was incubated with PT at 4 °C, the cells were washed and these cells were further incubated at 37 °C to enable the internalization of PT. Here, PTS1 was detected by the same antibody (Figure 4b, middle panel). In samples not treated with PT (control) no signal for PTS1 was detected (Figure 4b, lower panel). This indicates that the monoclonal PTS1 antibody preferably recognizes PTS1 if it is not in complex with the B subunit, suggesting that the epitope on PTS1 might be covered if PTS1 is part of the PT holotoxin. In context of the results from co-staining experiments with Golgi and ER markers that revealed almost no co-staining with Golgi and only partial co-localization with the ER, we hypothesize that most likely the main portion of PTS1, which is detected by this antibody by fluorescence microscopy, represents the cytosolic fraction of PTS1 because during all other steps of PT uptake, PTS1 is part of the holotoxin and therefore should not be detected in these experiments. Hence, the reduction of the PTS1 signal in the presence of CsA suggests that CsA might interfere with the uptake of PTS1 into the cytosol. From our results, however, we cannot determine which individual step of the retrograde uptake route of PT in cells is affected by CsA. However, since the cellular target molecules of CsA are the cytosolic Cyps and since it is known from other bacterial ADP-ribosylating toxins that the translocation of their enzyme domains from early endosomes into the cytosol is facilitated by Cyps and impaired by CsA, we hypothesize that in case of PT it might also be the translocation of PTS1 from the ER into the cytosol which is inhibited by CsA.

**Figure 5:**
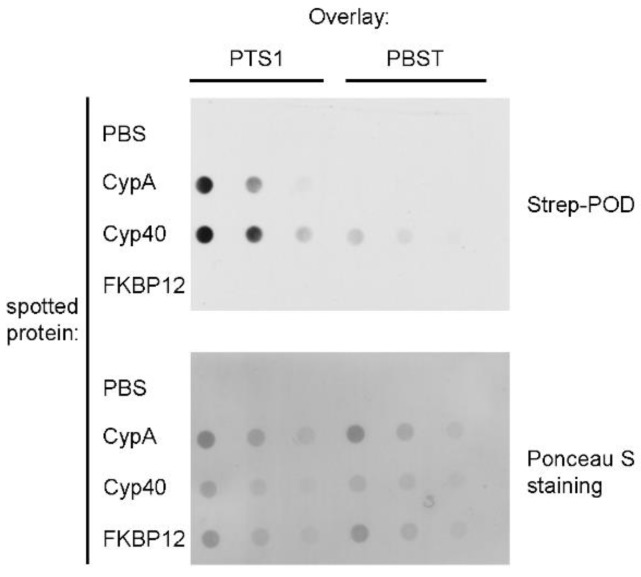
Purified CypA and Cyp40 proteins directly interact with PTS1 in vitro. Decreasing amounts of CypA and Cyp40 (1 µg, 0.5 µg and 0.25 µg) were spotted onto a membrane using the dot blot system. After blocking, the membrane was cut and probed with PTS1 (200 ng/mL) or PBST for control. After extensive washing, bound PTS1 was detected by anti-PTS1 antibody using the ECL system (upper panel). For further control, FKBP12 was spotted. Comparable amounts of spotted protein were confirmed by Ponceau S staining (lower panel).

CypA and Cyp40 specifically and directly interact with PTS1 in vitro. To analyse whether PTS1 interacts with Cyps directly, dot blotting was performed. Therefore, the purified recombinant Cyp isoforms CypA and Cyp40 were spotted onto a nitrocellulose membrane via vacuum-aspiration. Subsequently, an overlay with PTS1 was performed, which revealed that both Cyp isoforms interact in a direct and concentration-dependent manner with PTS1 in vitro (Figure 5). Only negligible signals on background level were detected if only buffer and not PTS1 was present during the overlay. For further control, another isoform of the PPIase family of FK506 binding proteins (FKBPs), FKBP12 was spotted. From earlier studies with enzyme components of other ADP-RTs, for example, of the clostridial binary toxins or the diphtheria toxin, it is known that FKBP12 does not interact with ADP-RTs and therefore serves as negative control in this experiment [27,31,37].

**Figure 6:**
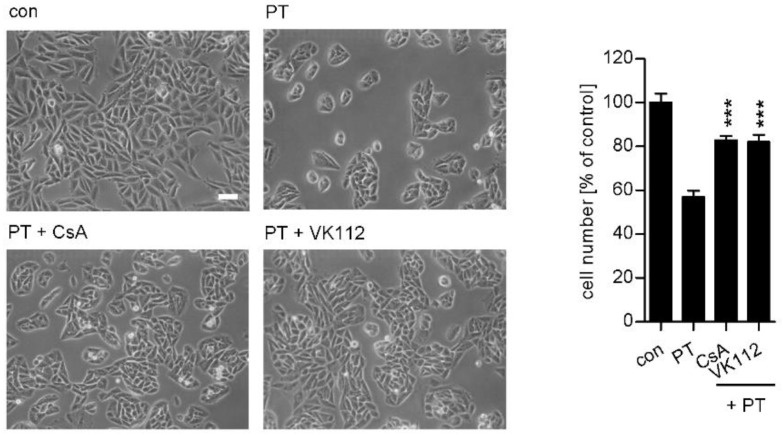
The non-immunosuppressive CsA-derivative VK112 protects CHO-K1 cells from intoxication with PT. CHO-K1 cells were pre-incubated with 10 µM VK112 or CsA for 30 min, or left untreated for control. PT (10 ng/mL) was added for 1 h in the presence or absence of the respective inhibitors. Subsequently, cells were incubated in fresh toxin- and inhibitor-free medium for 18 h. Pictures were taken after 18 h (left panel, white scale bar = 50 µm) and quantitative analysis of the total cell number of CHO-K1 cells is shown in the right panel. Values are normalized on control cells (*n* = 3 (3 different pictures from each sample were quantified), mean ± SD). Significance was tested by using the Student’s t test and values refer to samples treated with toxin only (*** *p* < 0.001).

The non-immunosuppressive CsA derivative VK112 also protects cells from PT-intoxication. Besides CsA, also a specifically designed non-immunosuppressive CsA derivative, VK112 [27,31,38], protected CHO-K1 cells from intoxication with PT (Figure 6). Pictures in Figure 6 reveal the reduced PT-induced clustering effect in the presence of VK112 compared to cells treated with PT only. Moreover, more cells were counted if cells were treated with PT in the presence of VK112 compared to cells treated with PT in the absence of VK112. Overall, the inhibitory effect of VK112 on PT intoxication was widely comparable to the effect of CsA.

Taken together, the results show that CsA as well as its tailored non-immunosuppressive derivative VK112 protect cells from intoxication with PT as demonstrated by morphology-based intoxication assays and biochemical analysis of the ADP-ribosylation status of Giα, the specific cytosolic substrate of PT. The direct interaction of CypA and Cyp40 with PTS1 and the reduced amount of PTS1 in the cytosol upon CsA treatment demonstrated by fluorescence microscopy suggests that Cyps play a functional role during translocation of PTS1 from the ER into the cytosol of the target cell.

## 3. Discussion

The sophisticated translocation of the A subunits of bacterial ADP-RTs from intracellular compartments into the cytosol of target cells has been investigated by us and others during past years (for review see [39,40]). It became evident that some specific host cell chaperones and protein folding helper enzymes facilitate the membrane transport of the A subunits that harbour the ADP-ribosyltransferase domains from early endosomes into the cytosol, as demonstrated for the family of clostridial binary toxins, which cause severe enterotoxicity in humans and animals and the diphtheria toxin, which causes symptoms of the upper respiratory tract disease diphtheria [27,28,29,31,37,41,42,43,44]. These toxins take a more direct route into the cytosol compared to PT. They are taken up by receptor-mediated endocytosis and then deliver their A subunit from acidified endosomes into the cytosol where they ADP-ribosylate their specific substrate molecules. However, as described for the cellular uptake of PT, a pre-requisite for translocation of these toxins is the at least partial unfolding of their A subunits [15,45]. We identified the heat shock proteins (Hsp)70 and Hsp90 as well as members of the PPIase families, namely FKBP51/52 but not FKBP12 and Cyps as crucial interaction partners of the A subunits of these ADP-RTs during their membrane translocation. Inhibition of the activity of the chaperones and/or protein folding helper enzymes by specific pharmacological inhibitors prevented the translocation of the A subunits into the cytosol and therefore protected cells from intoxication with clostridial binary toxins and the diphtheria toxin. Interestingly, all ADP-RTs investigated so far, including the *Photorhabdus luminescens* PTC3 and PTC5 toxins as well as different recombinant fusion toxins containing ADP-ribosyltransferase domains, require Hsp70, Hsp90, FKBPs and Cyps for their translocation into the cytosol [30,37,46]. In contrast, toxins displaying a different enzyme activity act independent of these factors [43,46,47]. This led to the hypothesis that the requirement of Hsp70, Hsp90, FKBPs and Cyps might be characteristic and specific for the intracellular membrane translocation of ADP-RTs.

Here, we demonstrated that the ADP-ribosylating PT requires Cyps for an efficient transport of its enzyme subunit PTS1 into the cytosol of target cells to modify its specific substrate Giα. Cyps are cytosolic proteins that facilitate the *cis/trans* isomerization of proline bonds, which is known as a rate-limiting step in protein folding. CsA specifically inhibits the PPIase activity of Cyps. Moreover, the complex of CsA and the most abundant isoform of Cyps, CypA (also known as Cyp18) inhibits the phosphatase calcineurin thereby interfering with activation of T lymphocytes and suppressing immune responses. CsA is a licensed drug used mainly after organ transplantation to prevent organ rejection [48]. Here, CsA revealed itself as a potent pharmacological inhibitor of PT intoxication, which reduced the amount of enzymatic active PTS1 in the cytosol and therefore diminished the ADP-ribosylation of Giα in cells.

Like many other severe diseases that is, diphtheria or cholera, whooping cough is a toxin-mediated disease [21,22]. Although the underlying mechanisms how PT precisely causes the disease still have to be elucidated, several facts indicate the importance of PT as the causative agent of whooping cough. Naturally occurring PT-deficient strains are extremely rare and no vaccine escape mutants have been observed so far. Vaccines that only contain detoxified PT are comparably effective to 3–5 component vaccines [49]. Although PT probably does not contribute to the coughing, it is known that PT causes leucocytosis in humans and animals. At the beginning of infection PT inhibits recruitment of neutrophils to the airways and impairs the anti-microbial activity of macrophages in the lung [21,50,51,52]. This supports the growth of *B. pertussis* in the airways. Later, PT levels correlate with increased inflammation and pathogenesis that cannot be resolved [53]. Thereby PT probably contributes to the long-lasting symptoms of whooping cough which has also been observed in several animal studies [21]. The importance of PT’s enzymatic activity was demonstrated by infecting volunteers with an engineered *B. pertussis* strain which expresses an enzymatically inactive version of PT [54]. Only minor respiratory symptoms that did not significantly differ from placebo groups were observed indicating that whooping cough is a disease mediated by an enzymatically active ADP-RT.

A requirement to achieve the PT-mediated effects is for PTS1 to reach the cytosol in an active that is, folded conformation. Since PTS1 has to be unfolded to escape from the ER using the ERAD pathway, refolding of PTS1 is required. Therefore, it seems plausible that protein folding helper enzymes such as Cyps not only facilitate the translocation of PTS1 from the ER into the cytosol but are also essentially involved in the refolding and stabilization of the thermally unstable PTS1 in an active conformation. The finding that different isoforms of Cyps directly interact with PTS1 supports this hypothesis.

The immunosuppressive effect of CsA constitutes a substantial drawback when considering CsA as a potential approach to treat whooping cough. The CsA derivative VK112 was designed to specifically target the PPIase site of Cyps without leading to the interaction with calcineurin and therefore lack the immunosuppressive effect [38]. Although the inhibitory efficacy on Cyps of VK112 is 7-fold less compared to the parent compound CsA, VK112 inhibited the intoxication of CHO-K1 cells with PT in the same concentration range as CsA. Earlier we showed that VK112 also inhibits the membrane translocation of clostridial binary toxins and the diphtheria toxin, thereby protecting cells from intoxication [27,31]. The effectiveness of a non-immunosuppressive derivative of CsA in preventing intoxication indicates the direct involvement of the active site of Cyps in this process. These findings open up the possibility to develop novel therapeutic strategies based on tailored inhibitors such as VK112 that not only target against symptoms of one disease but possibly against all diseases caused by ADP-RTs.

CsA and VK112 are specific inhibitors of Cyps, however distinction of different Cyp isoforms by CsA/VK112 is not possible. 18 different isoforms of Cyps are known and can be divided in single- and multi-domain Cyps [55]. CypA as a representative of the single-domain Cyps only comprises the PPIase domain. In contrast, the large isoform Cyp40 contains additional domains such as tetratricopeptide (TPR) domains which enable Cyp40 to interact with other host cell chaperones that possess a corresponding interaction site for example, Hsp90 [56,57]. This feature enables the formation of Hsp90-dependent multi-chaperone complexes that facilitate for example the folding and activation of steroid hormone receptors in the cell [58]. Further co-chaperones of this Hsp90-machinery include Hsp70 and FKBPs that are also required for the translocation of clostridial binary toxins and the diphtheria toxin. Interestingly, Hsp90 has been shown to facilitate the translocation not only of the clostridial binary toxins and the diphtheria toxin but also of the enzymatic subunit of the cholera toxin (CT), CTA1. CT and PT share high sequence and structural homology. Moreover, both toxins employ the retrograde uptake route including translocation from the ER into the cytosol. Therefore, we hypothesize that the translocation of ADP-RTs may be facilitated in a concerted manner by an Hsp90-based multi-chaperone complex including Hsp70, Cyps and FKPBs.

## 4. Materials and Methods

Cell culture and intoxication experiments. Chinese hamster ovary cells strain K1 (CHO-K1, Leibniz Institute DSMZ-German Collection of Microorganisms and Cell Cultures) were cultivated in DMEM and HAM’s F12 containing 5% heat-inactivated foetal calf serum, 1 mM sodium-pyruvate and Penicillin-Streptomycin (1:100). The cells were grown at 37 °C and 5% CO_2_. Cells were trypsinized and reseeded every two to three days for at most 15 to 20 times. For intoxication experiments cells were seeded in culture dishes and the specific pharmacological inhibitors CsA (inhibitor of cyclophilin activity, Sigma-Aldrich, Munich, Germany), VK112 (non-immunosuppressive CsA derivative, inhibitor of Cyps, synthesis performed as described before [38]) or BFA (disrupts Golgi apparatus, Sigma-Aldrich) were added for 30 min. For control, cells were left untreated. Then, PT (Sigma-Aldrich) was added for 1 h with subsequent removal and further incubation for 18 h in toxin- and inhibitor-free medium at 37 °C. The morphology of the cells was documented by using a Zeiss Axiovert 40CFL microscope with a Jenoptik ProgRes C10 CCD camera. To quantify the toxin-induced effects on the CHO-K1 cells the total number of cells per picture was manually counted using ImageJ (National Institutes of Health, Bethesda, MD, USA). Untreated control cells were set as 100% and values of other samples were calculated accordingly. The materials for cell culture experiments were obtained from TPP Techno Plastic Products.

Sequential ADP-ribosylation of Giα in lysates from toxin-treated cells. CHO-K1 cells were pre-incubated with the respective inhibitors and subsequently intoxicated with PT for given incubation periods. Cells were lysed in ADP-ribosylation buffer (0.1 mM Tris-HCL (pH 7.6), 20 mM DTT and 0.1 µM ATP) and protease inhibitor complete (Roche) as described earlier [11], followed by an incubation with 170 ng PTS1 (Aviva Systems Biology, Eching, Germany) and 10 µM biotin-labelled NAD^+^ (Trevigen, Wiesbaden-Nordenstadt, Germany) for 1 h at room temperature for an in vitro ADP-ribosylation of the Giα, which had not yet been ADP-ribosylated by PT during the intoxication. Samples were subjected to SDS-PAGE, blotted and ADP-ribosylated, that is biotin-labelled, Giα was detected with streptavidin-peroxidase (Strep-POD, Sigma-Aldrich) using the enhanced chemiluminescence (ECL) system. Equal amounts of protein were confirmed by Ponceau-S-staining and Hsp90 detection with a specific antibody (Santa Cruz, CA, USA).

Binding of PT to cells. For binding analysis, CHO-K1 cells were pre-incubated with 20 µM CsA or left untreated for control. Cells were cooled to 4 °C and PT was added for 40 min. Cells were washed twice with cold PBS and lysed. Samples were analysed by SDS-PAGE and Western Blotting. Cell-associated PT was detected by a specific monoclonal antibody against PTS1.

Immunofluorescence. For fluorescence microscopy, cells were seeded in ibidi 8 well µ slides and fixed after intoxication experiments with 4% paraformaldehyde (PFA), permeabilized with Triton-X 100 (0.4% in PBS), treated with Glycin (100 nM in PBS) and blocked with 5% skim milk powder or 10% normal goat serum and 1% BSA in PBST for 1 h at 37 °C. Samples were incubated with monoclonal anti-PTS1 antibody (Santa Cruz, 1:50 diluted in 10% NGS and 1% BSA in PBS-Tween (PBST)) for 1 h at 37 °C. After washing, fluorescence-labelled secondary antibody anti-mouse 568 (goat) (Invitrogen) was added (1:750 diluted in 10% NGS and 1% BSA in PBST) for 1 h at 37 °C. Nuclei were stained with Hoechst and F-actin with phalloidin-FITC (Sigma-Aldrich). For staining of the Golgi apparatus a polyclonal anti-GM130 antibody (Abcam, Cambridge, UK) in combination with anti-rabbit 488 secondary antibody (goat) (Invitrogen, Carlsbad, CA, USA) and for ER-staining a fluorescence labelled ER-tracker (Molecular Probes, Invitrogen) was used. Between the individual working steps, the cells were washed excessively with PBS. Fluorescence imaging was performed with the iMIC Digital Microscope (FEI Munich, Gräfelfing, Germany) using the Live Acquisition 2.6 software (FEI Munich) and processed with ImageJ 1.4.3.47 software (National Institutes of Health, Bethesda).

In vitro protein-protein interaction analysis using dot blot system. CypA [59], Cyp40 [27] and FKBP12 [60] were expressed and purified as described earlier. Recombinant purified CypA and Cyp40 were vacuum-aspirated onto a nitrocellulose membrane using the dot blot system (Bio-Rad). For control, PBS, FKBP12 were spotted. Ponceau S staining confirmed comparable amounts of spotted protein. The membrane was blocked with 5% skim milk powder in PBST and subsequently cut into two pieces to perform the overlay with PTS1 and for control with PBST. After extensive washing, the membranes were probed with anti-PTS1 antibody to detect the bound that is, interacting PTS1. The membranes were washed again and probed with an HRP-conjugated secondary antibody. Signals were detected using the ECL system.

## References

[1] Stein P.E., Boodhoo A., Armstrong G.D., Cockle S.A., Klein M.H., Read R.J. (1994). The crystal structure of pertussis toxin. Structure 1993.

[2] Tamura M., Nogimori K., Murai S., Yajima M., Ito K., Katada T., Ui M., Ishii S. (1982). Subunit structure of islet-activating protein, pertussis toxin, in conformity with the A-B model. Biochemistry.

[3] Locht C., Coutte L., Mielcarek N. (2011). The ins and outs of pertussis toxin. FEBS J..

[4] Weiss A.A., Johnson F.D., Burns D.L. (1993). Molecular characterization of an operon required for pertussis toxin secretion. Proc. Natl. Acad. Sci. USA.

[5] Armstrong G.D., Howard L.A., Peppler M.S. (1988). Use of glycosyltransferases to restore pertussis toxin receptor activity to asialoagalactofetuin. J. Biol. Chem..

[6] Hausman S.Z., Burns D.L. (1993). Binding of pertussis toxin to lipid vesicles containing glycolipids. Infect. Immun..

[7] Witvliet M.H., Burns D.L., Brennan M.J., Poolman J.T., Manclark C.R. (1989). Binding of pertussis toxin to eucaryotic cells and glycoproteins. Infect. Immun..

[8] El Bayâ A., Linnemann R., von Olleschik-Elbheim L., Robenek H., Schmidt M.A. (1997). Endocytosis and retrograde transport of pertussis toxin to the Golgi complex as a prerequisite for cellular intoxication. Eur. J. Cell Biol..

[9] Lippincott-Schwartz J., Yuan L.C., Bonifacino J.S., Klausner R.D. (1989). Rapid redistribution of Golgi proteins into the ER in cells treated with brefeldin A: Evidence for membrane cycling from Golgi to ER. Cell.

[10] Plaut R.D., Carbonetti N.H. (2008). Retrograde transport of pertussis toxin in the mammalian cell. Cell. Microbiol..

[11] Xu Y., Barbieri J.T. (1995). Pertussis toxin-mediated ADP-ribosylation of target proteins in Chinese hamster ovary cells involves a vesicle trafficking mechanism. Infect. Immun..

[12] Burns D.L., Manclark C.R. (1986). Adenine nucleotides promote dissociation of pertussis toxin subunits. J. Biol. Chem..

[13] Hazes B., Boodhoo A., Cockle S.A., Read R.J. (1996). Crystal structure of the pertussis toxin-ATP complex: A molecular sensor. J. Mol. Biol..

[14] Plaut R.D., Scanlon K.M., Taylor M., Teter K., Carbonetti N.H. (2016). Intracellular disassembly and activity of pertussis toxin require interaction with ATP. Pathog. Dis..

[15] Banerjee T., Cilenti L., Taylor M., Showman A., Tatulian S.A., Teter K. (2016). Thermal Unfolding of the Pertussis Toxin S1 Subunit Facilitates Toxin Translocation to the Cytosol by the Mechanism of Endoplasmic Reticulum-Associated Degradation. Infect. Immun..

[16] Hazes B., Read R.J. (1997). Accumulating evidence suggests that several AB-toxins subvert the endoplasmic reticulum-associated protein degradation pathway to enter target cells. Biochemistry.

[17] Pande A.H., Moe D., Jamnadas M., Tatulian S.A., Teter K. (2006). The pertussis toxin S1 subunit is a thermally unstable protein susceptible to degradation by the 20S proteasome. Biochemistry.

[18] Worthington Z.E.V., Carbonetti N.H. (2007). Evading the proteasome: Absence of lysine residues contributes to pertussis toxin activity by evasion of proteasome degradation. Infect. Immun..

[19] Bokoch G.M., Katada T., Northup J.K., Hewlett E.L., Gilman A.G. (1983). Identification of the predominant substrate for ADP-ribosylation by islet activating protein. J. Biol. Chem..

[20] Katada T., Ui M. (1982). Direct modification of the membrane adenylate cyclase system by islet-activating protein due to ADP-ribosylation of a membrane protein. Proc. Natl. Acad. Sci. USA.

[21] Carbonetti N.H. (2015). Contribution of pertussis toxin to the pathogenesis of pertussis disease. Pathog. Dis..

[22] Pittman M. (1984). The concept of pertussis as a toxin-mediated disease. Pediatr. Infect. Dis..

[23] Mattoo S., Cherry J.D. (2005). Molecular pathogenesis, epidemiology, and clinical manifestations of respiratory infections due to *Bordetella pertussis* and other *Bordetella subspecies*. Clin. Microbiol. Rev..

[24] Surridge J., Segedin E.R., Grant C.C. (2007). Pertussis requiring intensive care. Arch. Dis. Child..

[25] Domenech de Cellès M., Magpantay F.M.G., King A.A., Rohani P. (2016). The pertussis enigma: Reconciling epidemiology, immunology and evolution. Proc. Biol. Sci..

[26] WHO (2016). Pertussis vaccines: WHO position paper, August 2015—Recommendations. Vaccine.

[27] Ernst K., Langer S., Kaiser E., Osseforth C., Michaelis J., Popoff M.R., Schwan C., Aktories K., Kahlert V., Malesevic M. (2015). Cyclophilin-facilitated membrane translocation as pharmacological target to prevent intoxication of mammalian cells by binary clostridial actin ADP-ribosylated toxins. J. Mol. Biol..

[28] Kaiser E., Pust S., Kroll C., Barth H. (2009). Cyclophilin A facilitates translocation of the *Clostridium botulinum* C2 toxin across membranes of acidified endosomes into the cytosol of mammalian cells. Cell. Microbiol..

[29] Kaiser E., Kroll C., Ernst K., Schwan C., Popoff M., Fischer G., Buchner J., Aktories K., Barth H. (2011). Membrane translocation of binary actin-ADP-ribosylating toxins from *Clostridium difficile* and *Clostridium perfringens* is facilitated by cyclophilin A and Hsp90. Infect. Immun..

[30] Lang A.E., Ernst K., Lee H., Papatheodorou P., Schwan C., Barth H., Aktories K. (2014). The chaperone Hsp90 and PPIases of the cyclophilin and FKBP families facilitate membrane translocation of *Photorhabdus luminescens* ADP-ribosyltransferases. Cell. Microbiol..

[31] Schuster M., Schnell L., Feigl P., Birkhofer C., Mohr K., Roeder M., Carle S., Langer S., Tippel F., Buchner J. (2017). The Hsp90 machinery facilitates the transport of diphtheria toxin into human cells. Sci. Rep..

[32] Hewlett E.L., Sauer K.T., Myers G.A., Cowell J.L., Guerrant R.L. (1983). Induction of a novel morphological response in Chinese hamster ovary cells by pertussis toxin. Infect. Immun..

[33] Pootong A., Budhirakkul P., Tongtawe P., Tapchaisri P., Chongsa-nguan M., Chaicumpa W. (2007). Monoclonal antibody that neutralizes pertussis toxin activities. Asian Pac. J. Allergy Immunol..

[34] Fabre I., Fabre G., Lena N., Cano J.P. (1986). Kinetics of uptake and intracellular binding of Cyclosporine A in RAJI cells, in vitro. Biochem. Pharmacol..

[35] Bertault-Pérès P., Maraninchi D., Carcassonne Y., Cano J.P., Barbet J. (1985). Clinical pharmacokinetics of ciclosporin A in bone marrow transplantation patients. Cancer Chemother. Pharmacol..

[36] Shitara Y., Takeuchi K., Nagamatsu Y., Wada S., Sugiyama Y., Horie T. (2012). Long-lasting inhibitory effects of cyclosporin A, but not tacrolimus, on OATP1B1- and OATP1B3-mediated uptake. Drug Metab. Pharmacokinet..

[37] Kaiser E., Böhm N., Ernst K., Langer S., Schwan C., Aktories K., Popoff M., Fischer G., Barth H. (2012). FK506-binding protein 51 interacts with *Clostridium botulinum* C2 toxin and FK506 inhibits membrane translocation of the toxin in mammalian cells. Cell. Microbiol..

[38] Prell E., Kahlert V., Rücknagel K.P., Malešević M., Fischer G. (2013). Fine Tuning the Inhibition Profile of Cyclosporine A by Derivatization of the MeBmt Residue. ChemBioChem.

[39] Barth H., Ernst K., Gopalakrishnakone P., Stiles B., Alape-Girón A., Dubreuil J.D., Mandal M. (2016). Chaperones and ADP-Ribosylating Bacterial Toxins. Microbial Toxins.

[40] Ernst K., Schnell L., Barth H. (2017). Host Cell Chaperones Hsp70/Hsp90 and Peptidyl-Prolyl Cis/Trans Isomerases Are Required for the Membrane Translocation of Bacterial ADP-Ribosylating Toxins. Curr. Top. Microbiol. Immunol..

[41] Ernst K., Liebscher M., Mathea S., Granzhan A., Schmid J., Popoff M.R., Ihmels H., Barth H., Schiene-Fischer C. (2016). A novel Hsp70 inhibitor prevents cell intoxication with the actin ADP-ribosylating *Clostridium perfringens* iota toxin. Sci. Rep..

[42] Ernst K., Schmid J., Beck M., Hägele M., Hohwieler M., Hauff P., Ückert A.K., Anastasia A., Fauler M., Jank T. (2017). Hsp70 facilitates trans-membrane transport of bacterial ADP-ribosylating toxins into the cytosol of mammalian cells. Sci. Rep..

[43] Haug G., Leemhuis J., Tiemann D., Meyer D.K., Aktories K., Barth H. (2003). The host cell chaperone Hsp90 is essential for translocation of the binary *Clostridium botulinum* C2 toxin into the cytosol. J. Biol. Chem..

[44] Haug G., Aktories K., Barth H. (2004). The host cell chaperone Hsp90 is necessary for cytotoxic action of the binary iota-like toxins. Infect. Immun..

[45] Haug G., Wilde C., Leemhuis J., Meyer D.K., Aktories K., Barth H. (2003). Cellular uptake of *Clostridium botulinum* C2 toxin: Membrane translocation of a fusion toxin requires unfolding of its dihydrofolate reductase domain. Biochemistry.

[46] Dmochewitz L., Lillich M., Kaiser E., Jennings L.D., Lang A.E., Buchner J., Fischer G., Aktories K., Collier R.J., Barth H. (2011). Role of CypA and Hsp90 in membrane translocation mediated by anthrax protective antigen. Cell. Microbiol..

[47] Zornetta I., Brandi L., Janowiak B., Dal Molin F., Tonello F., Collier R.J., Montecucco C. (2010). Imaging the cell entry of the anthrax oedema and lethal toxins with fluorescent protein chimeras. Cell. Microbiol..

[48] Galat A. (2003). Peptidylprolyl cis/trans isomerases (immunophilins): Biological diversity-targets-functions. Curr. Top. Med. Chem..

[49] Thierry-Carstensen B., Dalby T., Stevner M.A., Robbins J.B., Schneerson R., Trollfors B. (2013). Experience with monocomponent acellular pertussis combination vaccines for infants, children, adolescents and adults—A review of safety, immunogenicity, efficacy and effectiveness studies and 15 years of field experience. Vaccine.

[50] Carbonetti N.H. (2007). Immunomodulation in the pathogenesis of *Bordetella pertussis* infection and disease. Curr. Opin. Pharmacol..

[51] Carbonetti N.H., Artamonova G.V., Mays R.M., Worthington Z.E.V. (2003). Pertussis Toxin Plays an Early Role in Respiratory Tract Colonization by *Bordetella pertussis*. Infect. Immun..

[52] Kirimanjeswara G.S., Agosto L.M., Kennett M.J., Bjornstad O.N., Harvill E.T. (2005). Pertussis toxin inhibits neutrophil recruitment to delay antibody-mediated clearance of *Bordetella pertussis*. J. Clin. Investig..

[53] Connelly C.E., Sun Y., Carbonetti N.H. (2012). Pertussis toxin exacerbates and prolongs airway inflammatory responses during *Bordetella pertussis* infection. Infect. Immun..

[54] Thorstensson R., Trollfors B., Al-Tawil N., Jahnmatz M., Bergström J., Ljungman M., Törner A., Wehlin L., Van Broekhoven A., Bosman F. (2014). A phase I clinical study of a live attenuated *Bordetella pertussis* vaccine—BPZE1; a single centre, double-blind, placebo-controlled, dose-escalating study of BPZE1 given intranasally to healthy adult male volunteers. PLoS ONE.

[55] Schiene-Fischer C. (2015). Multidomain Peptidyl Prolyl cis/trans Isomerases. Biochim. Biophys. Acta.

[56] Li J., Buchner J. (2013). Structure, function and regulation of the hsp90 machinery. Biomed. J..

[57] Pratt W.B., Toft D.O. (2003). Regulation of signaling protein function and trafficking by the hsp90/hsp70-based chaperone machinery. Exp. Biol. Med..

[58] Li J., Soroka J., Buchner J. (2012). The Hsp90 chaperone machinery: Conformational dynamics and regulation by co-chaperones. Biochim. Biophys. Acta.

[59] Fanghänel J., Fischer G. (2003). Thermodynamic characterization of the interaction of human cyclophilin 18 with cyclosporin A. Biophys. Chem..

[60] Edlich F., Weiwad M., Wildemann D., Jarczowski F., Kilka S., Moutty M.-C., Jahreis G., Lücke C., Schmidt W., Striggow F. (2006). The specific FKBP38 inhibitor *N*-(*N*′,*N*′-dimethylcarboxamidomethyl)cycloheximide has potent neuroprotective and neurotrophic properties in brain ischemia. J. Biol. Chem..

